# MOG-IgG in primary and secondary chronic progressive multiple sclerosis: a multicenter study of 200 patients and review of the literature

**DOI:** 10.1186/s12974-018-1108-6

**Published:** 2018-03-19

**Authors:** S. Jarius, K. Ruprecht, J. P. Stellmann, A. Huss, I. Ayzenberg, A. Willing, C. Trebst, M. Pawlitzki, A. Abdelhak, T. Grüter, F. Leypoldt, J. Haas, I. Kleiter, H. Tumani, K. Fechner, M. Reindl, F. Paul, B. Wildemann

**Affiliations:** 1 Molecular Neuroimmunology Group, Department of Neurology, University of Heidelberg, Heidelberg, Germany; 2 Department of Neurology, Charité – University Medicine Berlin, Berlin, Germany; 3 Institut für Neuroimmunologie und Multiple Sklerose (INIMS), Zentrum für Molekulare Neurobiologie Hamburg, Universitätsklinikum Hamburg-Eppendorf, Hamburg, Germany; 4 Klinik und Poliklinik für Neurologie, Universitätsklinikum Hamburg-Eppendorf, Hamburg, Germany; 5 Department of Neurology, University of Ulm, Ulm, Germany; 6 Department of Neurology, Ruhr University Bochum, Bochum, Germany; 7 Department of Neurology, Hannover Medical School, Hanover, Germany; 8 Department of Neurology, Otto von Guericke University Magdeburg, Magdeburg, Germany; 9 Department of Neurology and Institute of Laboratory Medicine, University Hospital Schleswig-Holstein, Kiel, Germany; 10 Marianne-Strauß-Klinik, Behandlungszentrum Kempfenhausen für Multiple Sklerose Kranke, Berg, Germany; 11 Specialty Clinic of Neurology Dietenbronn, Schwendi, Germany; 12 Institute of Experimental Immunology, affiliated to Euroimmun AG, Lübeck, Germany; 13 Clinical Department of Neurology, Medical University of Innsbruck, Innsbruck, Austria; 14 NeuroCure Clinical Research Center, Charité - Universitätsmedizin Berlin, corporate member of Freie Universität Berlin, Humboldt-Universität zu Berlin, and Berlin Institute of Health, Berlin, Germany; 15 Experimental and Clinical Research Center, Max Delbrück Center for Molecular Medicine, Berlin, Germany; 16 Otto Meyerhof Center, Im Neuenheimer Feld 350, 69120 Heidelberg, Germany

**Keywords:** Myelin oligodendrocyte glycoprotein (MOG), Antibodies, Immunoglobulin G, MOG-IgG, Primary chronic progressive MS (PPMS), Secondary chronic progressive MS (SPMS), Neuromyelitis optica spectrum disorders (NMOSD)

## Abstract

**Background:**

Antibodies to human full-length myelin oligodendrocyte glycoprotein (MOG-IgG) as detected by new-generation cell-based assays have recently been described in patients presenting with acute demyelinating disease of the central nervous system, including patients previously diagnosed with multiple sclerosis (MS). However, only limited data are available on the relevance of MOG-IgG testing in patients with chronic progressive demyelinating disease. It is unclear if patients with primary progressive MS (PPMS) or secondary progressive MS (SPMS) should routinely be tested for MOG-IgG.

**Objective:**

To evaluate the frequency of MOG-IgG among patients classified as having PPMS or SPMS based on current diagnostic criteria.

**Methods:**

For this purpose, we retrospectively tested serum samples of 200 patients with PPMS or SPMS for MOG-IgG using cell-based assays. In addition, we performed a review of the entire English language literature on MOG-IgG published between 2011 and 2017.

**Results:**

None of 139 PPMS and 61 SPMS patients tested was positive for MOG-IgG. Based on a review of the literature, we identified 35 further MOG-IgG tests in patients with PPMS and 55 in patients with SPMS; the only reportedly positive sample was positive just at threshold level and was tested in a non-IgG-specific assay. In total, a single borderline positive result was observed among 290 tests.

**Conclusion:**

Our data suggest that MOG-IgG is absent or extremely rare among patients with PPMS or SPMS. Routine screening of patients with typical PPMS/SPMS for MOG-IgG seems not to be justified.

## Background

Antibodies to human full-length myelin oligodendrocyte glycoprotein (MOG-IgG) as detected by cell-based assays have recently been implicated in the pathogenesis of central nervous system (CNS) demyelination [1]. Most adult MOG-IgG-positive patients present with optic neuritis (ON), myelitis or brainstem encephalitis, though supratentorial brain lesions and epileptic seizures may occur as well [2–9]. In addition, MOG-IgG has been demonstrated in (mostly paediatric) patients with acute disseminated encephalomyelitis. MOG-IgG-related encephalomyelitis (MOG-EM) is now considered by many experts a disease entity in its own right, pathogenetically distinct from both classic multiple sclerosis (MS) and aquaporin-4 (AQP4)-IgG-positive neuromyelitis optic spectrum disorders [10]. So far, MOG-IgG has been almost exclusively reported in patients with monophasic or relapsing-remitting acute disease. However, as a major limitation, many previous studies had explicitly excluded patients with chronic progressive demyelination. It is therefore possible that MOG-IgG has so far been overlooked in patients with chronic progressive MS. To obtain more definite data on the role of MOG-IgG in chronic progressive CNS demyelination, we decided to test a large cohort of patients previously diagnosed with primary progressive MS (PPMS) or secondary progressive MS (SPMS) for MOG-IgG.

## Methods

Serum samples from 200 patients with chronic progressive MS according to the 2010 McDonald criteria, comprising 139 with PPMS and 61 with SPMS, were retrospectively tested for MOG-IgG. Human embryonic kidney (HEK) 293 cells transfected with full-length human MOG were used as antigenic substrate in combination with control cells as previously described [4, 11]. Samples yielding both a titer of ≥ 1:10 in the fixed cell-based assay and a titer of ≥ 1:160 in the live cell-based assay were considered positive [4, 11]. The sex ratio was 1:1.2 (female to male) in the total cohort, 1:1.9 in the PPMS subgroup, and 1:0.36 in the SPMS subgroup. The median age at the time of testing was 48 years (range 18–77) in the total cohort, 51 (31–77) among patients with PPMS, and 47 (22–74) among patients with SPMS. The median expanded disability status scale (EDSS) score was 4.5 (range 1.5–9) in the total cohort, 4 (1.5–9) among patients with PPMS, and 6.5 (2–9) among patients with SPMS. The median disease duration at the time of blood sampling was 7.9 years (range 1–50.7) in the total cohort, 6 (range 1–36) in the PPMS subgroup, and 17 (range 1–50.7) in the SPMS subgroup. Data on treatment at the time of blood sampling were available from 188/200 (98%) patients; most patients (151/188 or 80.3%) were not treated with immunosuppressive drugs or steroids at the time of sampling. The study was approved by the institutional review boards of the participating centers. The patients gave written informed consent or were tested in an anonymised fashion as required by the institutional review board of the University of Heidelberg. Samples were stored at − 80 °C prior to testing. Sixteen MOG-IgG-positive serum samples from previously reported patients with ON and/or myelitis were used as positive controls [2–5], including 13 samples that had previously yielded low-titer results in the live-cell assay (1:160–1:320). In addition, we performed a review of the entire literature on MOG-IgG published in English in journals indexed in the PubMed database of the US Library of Science at the US National Institutes of Health between 2011 and 2017.

## Results

None of 200 patients with primary (*N* = 139) or secondary (*N* = 61) chronic progressive MS was positive for MOG-IgG. All positive controls were correctly detected. Based on a review of the English language literature, 35 additional tests for MOG-IgG in patients with PPMS and 55 in patients with SPMS were identified (Table 1). The only reportedly MOG-IgG-positive case we could identify in the literature—a patient previously diagnosed with SPMS—was positive just at threshold level when tested in a semiquantitative assay [11]. Considering the 200 patients tested in the present study and the 90 reported in the previous literature, MOG-IgG has been detected in 0/174 patients with PPMS and in 1/116 patients with SPMS, with the only reportedly positive sample having yielded a borderline result (Table 1). Moreover, the latter sample was positive in a semiquantitative assay employing a non-Fc specific secondary anti-IgG antibody recognizing both heavy and light chains, leaving the possibility that the patient was in fact positive for MOG-IgM antibodies of unclear diagnostic relevance rather than MOG-IgG antibodies.

**Table 1:** MOG-IgG in patients with PPMS and SPMS as found in the present study and as reported in the literature

	PPMS	SPMS
Jarius et al., present study	139, none positive for MOG-IgG	61, none positive for MOG-IgG
Jarius et al., J Neuroinflammation 2016 [4]	5, none positive for MOG-IgG	11, none positive for MOG-IgG
Martinez-Hernandez et al., JAMA Neurol 2015 [16]	10, none positive for MOG-IgG	9, none positive for MOG-IgG
Höftberger et al., Mult Scler 2015 [17]	10, none positive for MOG-IgG	10, none positive for MOG-IgG
Mader et al., J Neuroinflammation 2011 [11]	8, none positive for MOG-IgG	19, one borderline positive for MOG-IgG (1:160)
Ramanathan et al., Neurol Neuroimmunol Neuroinflamm 2014 [18]	2, none positive for MOG-IgG	6, none positive for MOG-IgG
*Total*	*0/174*	*1/116 (borderline result)*

## Discussion

Given the potential prognostic and therapeutic consequences of a chronic progressive disease course, addressing the question of whether MOG-IgG can be associated with chronic progressive demyelination is of high clinical relevance. Our data indicate that MOG-IgG is present only extremely rarely or not at all in adult patients diagnosed with PPMS or SPMS (one borderline result among 290 tests [0.3%]; see Table 1 for details).

The very low frequency of MOG-IgG among patients diagnosed with PPMS/SPMS on the one hand and the lack of standardized assays for MOG-IgG testing and the limited specificity of immunoassays in general on the other hand bears the significant risk of an unfavourable ratio of false-positive to true-positive results (a risk generally attached to screening of large cohorts for very rare markers [12, 13]). Based on the 0–0.3% positivity rate among PPMS/SPMS patients observed in this study, the number of false-positive results might even outnumber the number of true-positive results if all PPMS/SPMS patients were to be tested for MOG-IgG and assay specificity were to be less than 99.7–100%. Therefore, we advise against routine screening for MOG-IgG in patients with PPMS or SPMS. Instead, we recommend limiting MOG-IgG testing in patients with PPMS or SPMS to those with clinical and/or paraclinical findings considered suggestive of MOG-IgG-related encephalomyelitis, including, for example, predominant attacks of ON or myelitis, longitudinally extensive optic nerve or myelitis lesions, typical brain magnetic resonance imaging (e.g. normal supratentorial MRI; or no Dawson finger lesion, no juxtacortical U fibre lesion, no ovoid/round lesion adjacent to a lateral ventricle, and no lesion in the temporal lobe), or cerebrospinal fluid findings that are atypical for MS (e.g. negative oligoclonal bands, neutrophilic pleocytosis, or white cell count >50/µl) [2, 3, 14]. Finally, given that a progressive disease course seems to be rare in MOG-EM [3] and thus atypical, confirming a positive test result in patients with PPMS/SPMS using a second, methodologically independent assay or, if that is not possible, by testing of follow-up samples (ideally taken during acute relapse [3] and/or during treatment-free intervals) is advisable.

Finally, it should be taken into account that it can be difficult in clinical practice to distinguish between a secondary chronic progressive disease course and disability progression resulting from protracted attacks with incomplete remission or from mild attacks, some of which may be just below the patient’s threshold of attention. In case of doubt, MOG-IgG testing may therefore also be justified in selected patients with suspected chronic progressive disease, especially if the findings are otherwise suggestive of MOG-EM. However, confirmation of a positive result should be sought as outlined above.

We recognize that our study has potential limitations: First, all of our patients were adults. While to the best of our knowledge no paediatric patients with a progressive course and confirmed MOG-IgG-positive serostatus have yet been reported, further studies are certainly needed before any recommendations regarding MOG-IgG testing in children with chronic progressive CNS demyelinating disease can be made. Second, we report data from a European cohort; to formally rule out that genetic factors play a role, testing of other, e.g. Asian, populations seems advisable due to potential genetic differences. Third, treatment effects may influence antibody titers. However, the vast majority of patients analysed here were not treated with immunosuppressive drugs at the time of blood sampling, and MOG-IgG was also absent in all 151 patients not treated with immunosuppressants or steroids. Fourth, assessing disease activity in progressive MS according to current recommendations [15] requires monitoring of MRI activity over time, since relapses are typically rare or missing. As standardized MRI data were not available for the present cohort, we cannot formally exclude a potential effect of disease activity. However, it is likely that disease activity in our cohort was at least similar to that in the general PPMS/SPMS population, since unselected, consecutive patients were tested and since the sample size was high. Given that all patients were recruited at tertiary centers and that patients with inactive, stable disease are less likely to be seen at tertiary centers, disease activity may have been even higher than in the general PPMS/SPMS population. On the other hand, we count the multicenter design, which helped to lower the risk of selection bias, the additional literature review, and the very large number of patients tested among the strengths of this study.

## Conclusion

In summary, our results argue against a major role of MOG-IgG in patients with primary or secondary progressive demyelination and may prove useful for future recommendations on clinical indications for MOG-IgG testing.

## Abbreviations

AQP4: Aquaporin-4
CNS: Central nervous system
EM: Encephalomyelitis
IgG: Immunoglobulin G
MOG: Myelin oligodendrocyte glycoprotein
MS: Multiple sclerosis
PPMS: Primary progressive MS
SPMS: Secondary progressive MS
